# Hereditary Colorectal Cancer Syndromes: Molecular Genetics and Precision Medicine

**DOI:** 10.3390/biomedicines10123207

**Published:** 2022-12-10

**Authors:** Liuxiang Chen, Liansong Ye, Bing Hu

**Affiliations:** Department of Gastroenterology, West China Hospital, Sichuan University, Chengdu 610041, China

**Keywords:** hereditary colorectal cancer syndromes, predisposing genes, prevention strategies

## Abstract

Colorectal cancer (CRC) is the third most commonly diagnosed cancer worldwide. Hereditary CRC syndromes account for approximately 5–10% of all CRC, with a lifetime risk of CRC that approaches 50–80% in the absence of endoscopic or surgical treatment. Hereditary CRC syndromes can be phenotypically divided into polyposis and non-polyposis syndrome, mainly according to the conditions of polyps. The typical representatives are familial adenomatous polyposis (FAP) and Lynch syndromes (LS), respectively. Over the past few decades, molecular genetics enhanced the discovery of cancer-predisposing genes and revolutionized the field of clinical oncology. Hereditary CRC syndromes have been a key part of this effort, with data showing that pathogenic variants are present in up to 10% of cases. Molecular phenotypes of tumors can not only help identify individuals with genetic susceptibility to CRC but also guide the precision prevention and treatment for the development of CRC. This review emphasizes the molecular basis and prevention strategies for hereditary CRC syndromes.

## 1. Introduction

Hereditary colorectal cancer (CRC) syndromes exhibit different inheritance patterns and phenotypic features. The expansion of family registries and advances in genomic technologies have led to the discovery of multiple genes for specific hereditary syndromes, facilitating clinical diagnosis and treatment. Genetic susceptibility is associated with 2–8% of all CRCs and 1/5 of CRCs diagnosed before the age of 50 due to pathogenic germline variation in high-risk cancer genes [1,2,3,4,5]. In hereditary CRC syndromes, adenomatous polyposis syndrome accounts for ~1% and is strongly associated with *APC* (dominant inheritance) and *MUTYH* (recessive inheritance); Lynch syndrome (LS) accounts for 2–3% and is associated with germline or epistatic mutations in the DNA mismatch repair (MMR) genes *MLH1*, *MSH2*, *MSH6*, and *PMS2*; hamartomatous polyposis syndrome (<0.1%) is caused by mutations in *SMAD4*, *BMPR1A*, *STK11*, and *PTEN*. Nowadays, the understanding of genetic susceptibility to CRC is more extensive. Continued molecular genetic studies help to understand the nature of mutations in cancer susceptibility genes and facilitate the therapeutic task of individualized medicine. Therefore, the following article outlines the characteristics of hereditary CRC syndromes, the susceptibility genes proposed in recent years, and the associated chemopreventive and immunotherapeutic strategies.

## 2. Overview of Hereditary CRC Syndromes

### 2.1. Adenomatous Polyposis Syndromes

**Familial adenomatous polyposis (FAP)** is the most common adenomatous polyposis syndrome. FAP is a rare autosomal dominant disorder characterized by multiple adenomatous polyps of the gastrointestinal mucosa and various extraintestinal lesions, with a 100% lifetime risk of CRC if not treated [6] (Table 1). FAP can be classified into classic and attenuated types. Attenuated FAP (AFAP) is a milder form, with a delayed age of onset, fewer polyps (0–100), a lower overall risk of CRC (60–80%), and mostly in the right hemicolon. Early identification and management of FAP are essential due to the nearly 100% lifetime risk of CRC without intervention. Germline mutations in the *APC* (MIM: 611731) gene can be detected in more than 90% of FAP cases. *APC* testing should be taken into consideration when one of the following occurs: (1) more than 20 adenomas in the colorectum over the course of the patient’s lifetime, (2) a family history of FAP, or (3) 10 cumulative adenomas discovered after a colonoscopy. Screening colonoscopy has been proven to lower the risk of CRC in patients with FAP until the polyp burden is unmanageable by endoscopic methods. In families with classic FAP, a high-quality colonoscopy is recommended beginning between the ages of 10–15 years and repeated every year if an *APC* variant is identified. Early colonoscopy initiation may be taken into consideration for patients who have a family history of CRC with very early onset. Due to the attenuated character of AFAP, colonoscopy can be started at a slightly later age (late teens) and repeated every 1–2 years [6,7]. Extraintestinal manifestations in FAP involve duodenal adenomas, desmoids, thyroid tumors, and central nervous system tumors [8,9]. Consequently, monitoring and treating extracolonic lesions is also extremely crucial.

**Polymerase proofreading-associated polyposis (PPAP)** has been identified as a novel adenomatous polyposis syndrome, which is an autosomal dominant diseasecaused by germline pathogenic variants in the exonuclease (proofreading) domains of *POLE* (MIM: 174762) and *POLD1* (MIM: 174761) [10]. Although the clinical phenotype has not been established, the available evidence showed that PPAP has a high-penetrant susceptibility to polyposis, CRCs, and other extracolonic tumors.

**MUTYH-associated polyposis (MAP)** is considered to be the second most common adenomatous polyposis syndrome. MAP is an autosomal recessive condition with high penetration and is linked to biallelic germline variants in the DNA glycosylase gene *MUTYH* (MIM: 604933). The identification of *MUTYH* lays the foundation for the base excision repair (BER) pathway in polyposis and CRC. Carriers of biallelic *MUTYH* variants exhibit a high lifetime risk of CRC, while it is debatable whether patients with a monoallelic mutation have an increased genetic susceptibility to CRC (1.5–2 fold) [11,12]. Patients with MAP display a very broad and diverse clinical spectrum, which is generally more compared to AFAP. Therefore, endoscopic surveillance is recommended from age 18 with a 1–2 years interval, according to the findings [13].

Recently, two novel adenomatous polyposis-predisposing syndromes with autosomal recessive inheritance have been proposed. *NTHL1* (MIM: 602656) is the second DNA glycosylase gene identified for the BER pathway [14]. Biallelic mutations of *MSH3* (MIM: 600887), an MMR gene not linked to LS, were detected in families with polyposis and gastrointestinal malignancy, thereby identifying as another recessive subtype of colorectal adenomatous polyposis syndromes [15].

**Constitutional MMR deficiency syndrome (CMMRD)** is an uncommon, inherited cancer predisposition syndrome caused by biallelic germline pathogenic variants in four MMR genes, *MLH1* (MIM: 120436), *MSH2* (MIM: 609309), *MSH6* (MIM: 600678), and *PMS2* (MIM: 600259). Such cases with biallelic germline mutations in the MMR gene are distinct from classic LS. Patients with CMMRD have a higher risk of developing cancers as early as childhood and adolescence. It was estimated that 80% of patients experience their first cancer before 18 years old [16].

### 2.2. Hamartomatous Polyposis Syndromes

A hamartoma is a disordered and uncontrolled proliferation of normal cells. Based on this, the hamartomatous polyposis syndromes are distinguished from the adenomatous polyposis syndromes. Even though there is no direct evidence, hamartomatous polyposis syndromes have an estimated prevalence of 1/100,000 [17]. The types of gastrointestinal hereditary hamartomatous syndromes mainly include Peutz–Jeghers syndrome (PJS), PTEN-hamartoma tumor syndrome (PHTS), and juvenile polyposis syndrome (JPS). The evaluation criteria have been created by National Comprehensive Cancer Network (NCCN). The patient’s medical history, a skin-focused physical examination, and the feature of gastrointestinal polyps should be taken into consideration during the diagnostic process. Surprisingly, colonoscopy almost reduces the risk of CRC to the level of the general population.

**Peutz–Jeghers Syndrome (PJS)** is a rare inherited autosomal dominant syndrome known as mucocutaneous pigmentations because nearly all individuals exhibit melanotic macules or “spots” on their lips, buccal mucosa, fingertips, and toe tips. Hamartomatous polyps are mainly found in the small intestine (60–90%), colon (50–64%), or extraintestinal organs such as the gallbladder or bladder [18]. PJS is linked to an increased risk of CRC, and the majority of cases result from mutations in the tumor suppressor gene *LKB1/STK11* (MIM: 602216).

**Juvenile polyposis syndrome (JPS)** is also an inherited autosomal dominant syndrome driven by mutations in either *BMPR1A* (MIM: 601299) or *SMAD4* (MIM: 600993) [19]. Patients with JPS are characterized by multiple hamartomatous polyps in the gastrointestinal tract, primarily colon polyps, occasionally stomach polyps, and infrequently small intestine polyps. There is no extra risk of non-gastrointestinal cancer reported in JPS.

**PTEN-hamartoma tumor syndrome (PHTS)** is a disorder caused by mutations in the tumor suppressor gene *PTEN* (MIM: 601728) and is linked to increased risk for cancer in multiple organs, including the breast, thyroid, kidney, and skin. The gastrointestinal manifestations of PHTS are mainly gastrointestinal hamartomatous polyps. Although there is a moderate risk of CRC, colonoscopic surveillance can be delayed until after the age of 35. Notably, although most cases of PHTS correspond to Cowden syndrome (CS), PHTS also includes Bantayan–Riley–Ruvalcaba syndrome (BRRS).

### 2.3. Other Polyposis Syndromes

**Serrated polyposis syndrome (SPS)** is a hereditary condition known as multiple serrated polyps throughout the colorectum, including hyperplastic polyps, sessile serrated lesions, sessile serrated lesion with dysplasia, and conventional serrated adenomas [20]. Patients with SPS have an increased risk (16–29%) of CRC [21]. *RNF43* (MIM: 612482), along with *BRAF* (MIM: 164757) mutation, has been identified in rare cases of serrated polyposis [22]. However, high-penetrance germline mutations have not been found yet.

**Mixed polyposis syndrome (MPS)** is a rare autosomal dominant disease and is characterized by the presence of multiple histologic polyps, including adenomas, hamartomas, and serrated lesions, with an increased risk of CRC. Some affected people have been found to have *GREM1* (MIM: 603054) germline variants [23,24], while the genetic basis of most MPS families is still unclear, and there are insufficient data to prove the optimal monitoring interval.

### 2.4. Non-Polyposis Syndromes

**LS** is the most common hereditary non-polyposis syndrome, accounting for 2–4% of all CRCs, and was initially identified by Lynch in 1996 [25,26]. LS is an autosomal dominant condition caused by a germline mutation in one of the DNA MMR genes *MLH1*, *MSH2*, *MSH6*, and *PMS2*, which preserve genome integrity by postreplicative proofreading and editing. LS has a significantly higher risk of CRC, and an estimated 60–80% of MMR mutations will lead to CRC progression [27]. Patients with LS also have an increased risk of developing extra-colon cancers such as endometrial, ovarian, stomach, and small intestine cancers, especially if they have mutations in the *MLH1*, *MSH2*, and *MSH6* genes.

In contrast to polyposis syndromes, LS does not exhibit any characteristic endoscopic signs, making it a particularly difficult condition to diagnose. The Amsterdam criteria, which had been developed in 1991, served as the basis for the initial diagnosis [28]. These criteria concentrated on age at onset and family history, which could help to select families for genetic identification research. Following the discovery of a germline mutation in an MMR gene, Bethesda guidelines further use microsatellite instability (MSI) to select patients for genetic testing [29]. Neither of these criteria is optimal in identifying LS cases, prompting the recommendation to offer genetic testing to all newly diagnosed CRC patients [30,31]. All people with a molecular diagnosis of LS should have a colonoscopy starting at 20–25 [7]. Vasen et al. tracked 205 LS families and found a decreased incidence of CRC with colonoscopy intervals of 1–2 years vs. 2–3 years [32]. NCCN recommendations urge surveillance colonoscopy every 1–2 years for mutation carriers [33]. Given the decreased cancer risk in MSH6 and PMS2 mutation carriers, surveillance at age 25–30 may be considered [34].

**Familial Colorectal Cancer Type X (FCCTX)** is a kind of hereditary non-polyposis CRC with autosomal dominant inheritance, which also meets Amsterdam criteria Ⅱ. Unlike LS, its MMR gene has no germline mutation and shows microsatellite stability (MSS) [35]. This genetic difference is important because it determines the management strategies. The age at diagnosis of FCCTX is later than that of LS (approximately 40 years), and they do have a 2-fold increased risk compared to the general population. Although there is currently no standard follow-up protocol for monitoring and intervention, patients are recommended to get screening at the age of 40, but not before. 

## 3. Predisposing Genes for Hereditary CRC Syndromes

Linkage analysis was the dominant tool of gene discovery until the Millenium shift and was used to discover genes of hereditary CRC syndromes [36]. Most of the more than 100 known cancer susceptibility genes were found by a mendelian-type family linkage approach, and the majority of the 10 high-risk genes for CRC susceptibility have also been identified by this method [36,37]. To this day, technologies such as whole-genome sequencing (WGS) and whole-exome sequencing (WES) have not only revolutionized tumor genomics but also made it an essential component of precision cancer therapy. The number of genes involved with inherited CRC syndrome predisposition is constantly growing.

### 3.1. Adenomatous Polyposis Syndromes

**Figure 1:**
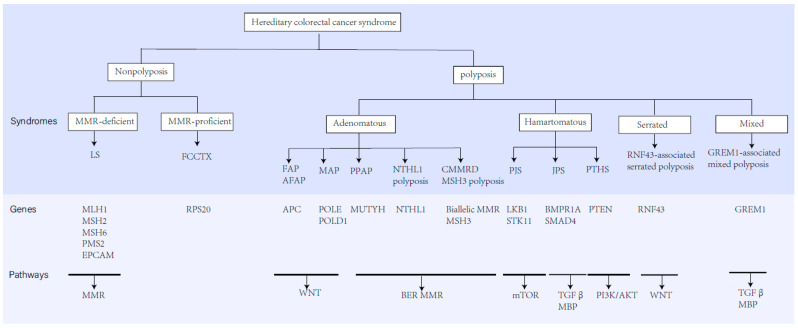
The classification, predisposing genes, inheritance, and related molecular pathways of hereditary CRC syndromes.

APC, located on chromosome 5q21, is a tumor suppressor gene for CRC [8,38]. The main pathogenic mechanism is disturbing the Wnt/β-catenin pathway. Mutated *APC* protein lacks the ability to degrade the β-catenin, and its cytoplasmic accumulation has been observed in FAP patients, which can be inhibited by the non-steroid anti-inflammatory drug (NSAIDs) sulindac [39] (Figure 1). Another consequence of *APC* mutations is chromosomal instability (CIN), which can lead to polyps being more likely to acquire a second strike [40]. Even though *APC* is a key gene for FAP or AFAP, about 10–30% of patients do not have *APC* variants [41]. Possible explanations for this may be mutations in other genes that have not yet been identified. As a result, numerous research sought to identify new genes that predispose to adenomatous polyposis syndromes.

In 2002, it was discovered that certain variations of the *MUTHY* gene were associated with an increased risk of developing numerous colorectal adenomas and carcinomas [42]. The *MUTHY* gene, located on chromosomes 1p32.1–p34.3, performs an important role in BER and has the ability to defend against DNA damage under oxidative stress. Mutations in *MUTYH* may lead to G:C→T: A reversal in the replication process [42,43]. In this way, *MUTYH* mutations may contribute the tumor formation and development. Next, some studies have made efforts to search for other variants in genes involved in BER. *NTHL1* is the second DNA glycosylase gene identified in the BER pathway and is thought to be a predisposing gene for polyposis and CRC [14]. The p.Gln90* homozygous nonsense variant in *NTHL1* was detected in initial families, and additional research has reported the compound heterozygous mutations. The prevalence of *NTHL1*-associated syndrome is still unclear, but it was estimated at 1:114,770 according to the prevalence in the European population, which is only one-fifth of MAP (1:19,079) [44]. As the deficiency of *NTHL1* can lead to polyposis and carcinoma, and the tumor phenotypes are broad spectrum, Grolleman et al. suggested that biallelic *NTHL1* mutation should be included in the surveillance guidelines for MAP [45]. Breast cancer screening is also recommended because of the moderate risk.

With the development of next-generation sequencing (NGS) technologies, WES and WGS have become the primary detecting method for susceptibility genes, and new candidate genes for CRC are constantly proposed. *POLE* and *POLD1* were the first two variants found by the combination of linkage analysis and WES [10]. Germline pathogenic mutations in *POLE* and *POLD1* were found to be linked to multiple polyposis and CRC [46]. *POLE* and *POLD1* are members of the B family of replicative and repair DNA polymerases and have a proofreading function. A Chinese study investigated 1392 patients mainly affected by CRC and proposed a potential carcinogenesis hypothesis mediated by *POLE/POLD1*. The results showed that *POLE/POLD1* variants carriers had a significantly high frequency of MMR variants and tumor mutational burden (TMB). *POLE* variants may affect cancer development through MMR, TGFβ, and RTK/RAS/RAF signaling pathways, and *POLD1* through MMR pathways [46].

To uncover further genes with high-penetrance causative mutations, Adam et al. employed exome sequencing leukocyte DNA from 102 individuals with unexplained adenomatous polyposis and found a recessive variant of colorectal adenomatous polyposis caused by biallelic *MSH3* germline mutations, an MMR gene unrelated to LS [15]. Similar to this, Olkinuora et al. discovered that the biallelic nonsense variant of DNA MMR gene *MLH3* (MIM: 604395) underlies a novel syndrome with susceptibility to FAP or AFAP [47]. The *AXIN2* (MIM:604025) gene is also a key regulator of β-catenin degradation in the Wnt pathway. Rivera et al. identified a novel missense variant in *AXIN2* in one family with AFAP, which lays the foundation for screening *AXIN2* in FAP patients negative for alterations in *APC* and *MUTYH* [48]. With the molecular genetics of hereditary CRC syndromes becoming increasingly clear, the moderate penetrance genes have become a new research hotspot. *GALNT12* (MIM#610290) controlled the initiation of mucin-type O-linked glycosylation, which plays an important role in adhesion, migration, and immune surveillance. Recent studies have demonstrated the relationship between *GALNT12* as a moderate penetrance gene for CRC susceptibility [48,49,50,51].

### 3.2. Non-Adenomatous Polyposis Syndromes

Germline mutations in the *LKB1/STK11* gene, located on chromosome 19p13.3, have been detected in 50–70% of patients with PJS [52,53]. *LKB1* can regulate cell polarity through PAR proteins and cell metabolism by the AMPK pathway. Additionally, *LKB1* deficiency can inhibit serine biosynthesis and lead to DNA damage. In this way, *LKB1* mutation may contribute to tumor formation and development. However, *LKB1* is no clear mutation hotspot because of many missense mutations and deletions occurring through the whole *LKB1* gene. For 80% of CS cases, a germline mutation in the *PTEN* gene has been identified as the culprit [54]. The *PTEN* gene is located on chromosome 10q23.3, and the *PTEN* protein has phosphatase activity. Since it is involved in the DNA repair and cell cycle checkpoint, *PTEN* deficiency has been reported to result in chromosomal instability. *PTEN* can negatively regulate the activity of the PI3K/AKT signaling pathway, thus inhibiting the occurrence and development of tumors. In total, 50–60% of JPS cases are caused by mutations in *SMAD4* and *BMPR1A*, and both of them are implicated in TGFβ signaling, which regulates cell proliferation and differentiation [19]. Although there are no clear mutations detected in the remaining 40%–50% of patients, the mutation in *ENG* (MIM: 131195) and *SMAD9* (MIM:603295) has been reported in a small number of patients, which are also involved in the TGFβ signaling. Furthermore, contiguous deletion of *BMPR1A* and PTEN is associated with severe JPS in infancy.

The genetic mechanism of MPS remains unclear, but duplication of 40 kb in the 3′ regions of the *SCG5* (MIM: 173120) gene and upstream region of *GREM1,* has been identified as the pathogenic mutation in families of Ashkenazi descent [23]. There is also evidence to suggest that the *ENG* genes are involved in hyperplasic mixed polyposis [55]. In 2014, Gala et al. performed WES in 20 SPS families, and first identified germline *RNF43* pathogenic variants as causative for SPS [56]. *RNF43* was a negative Wnt regulator, and the dependency of SPS on the Wnt signaling pathway was also demonstrated in the organoid cultures [22]. In these serrated polyps, somatic alteration including BRAF mutations and a high level of promoter methylation has been reported [22]. In spite of the fact that *GREM1* and *MUTYH* have also been reported for SPS, genetic testing is typically inconclusive [57].

### 3.3. Non-Polyposis Syndromes

For non-polyposis cases, this genetic susceptibility to CRC has traditionally been linked to germline mutations or epimutations in the DNA MMR genes. The MMR system corrects base mismatches, insertions, and losses that occur during DNA replication or exogenous damage. According to the genetic and clinical level, DNA MMR deficiency (dMMR) vs. proficiency (pMMR) classified hereditary non-polyposis syndromes into LS and FCCTX, respectively. More than 90% of LS-associated CRCs are associated with germline mutations in the DNA MMR genes, including *MLH1* (42%), *MSH2* (33%), *MSH6* (18%), and *PMS2* (7.5%) [58]. The dMMR would result in DNA repair defects and high microsatellite instability (MSI-H). Thus, MSI can be a genetic marker for tumors with dMMR system. *EPCAM* (MIM: 185535) gene mutation was also regarded as the main cause of LS, and *EPCAM* gene mutation accounts for 1–3% of all LS patients [59]. The loss of the *EPCAM* gene will result in the methylation of the MSH2 promoter, which will eventually render *MSH2* inactive and cause LS.

Although the genetic basis of FCCTX is still unclear compared to LS, several candidate genes have been proposed, including *GALNT12*, *RPS20* (MIM: 603682) and *BRF1* (MIM: 604902), *SEMA4A* (MIM: 607292), and *FAN1* (MIM: 613534) [60,61,62]. These genes may participate in protein glycosylation, ribosome biosynthesis, semaphoring signaling, DNA repair, or various other biological pathways.

## 4. Precision Medicine in Hereditary CRC Syndromes

### 4.1. Prophylactic Surgery

Surgery is the cornerstone of treatment for hereditary polyposis and non-polyposis syndromes and should be performed when CRC occurs or when the polyp burden cannot be managed by colonoscopy. In patients with FAP and other polyp syndromes, prophylactic surgery remains the gold standard for CRC prevention, but the timing varies. The most common surgical alternatives are total abdominal colectomy with an ileorectal anastomosis (IRA) or proctocolectomy with end ileostomy or restoration via an ileal pouch-anal anastomosis [9]. The determination of the surgery method is mainly based on the burden of colorectal polyps and the options of the patients. Patients with IRA retain an excessive risk of rectal cancer. Although studies prophylactic surgery has been shown to significantly reduce the risk of CRC in FAP, prophylactic surgery for LS is controversial. Some scholars believe that the risk of cancer in LS is as high as 80% and that prophylactic surgery can reduce the risk of CRC, whereas others argue against prophylactic surgery for MMR carriers due to incomplete dominant inheritance, the fact that 15–20% of LS patients do not develop CRC, and the fact that 6–20% of patients still develop CRC after colectomy. Therefore, prophylactic surgery may be considered for those who have difficulty with or refuse to undergo colonoscopic follow-up. For people with hamartomatous polyposis syndrome, preventative surgical resection is not advised because colonoscopic therapy may bring the risk of CRC down to that of the normal population.

### 4.2. Chemoprevention

The primary objective of hereditary CRC syndromes is cancer prevention. Research into the chemoprevention of hereditary CRC syndromes has been encouraged by the need for frequent surveillance procedures, necessary surgical intervention, and ongoing risk of disease progression. This treatment offers an alluring alternative to preventing the development of cancer in high-risk cancer syndromes. Although chemoprevention has been reported to be effective for duodenal and colonic polyps in FAP and for reducing the risk of cancer in LS, data from clinical trials are still sparse. An ideal preventive medication should be low toxicity, long-time effective, and easily accessible. Current research mainly focused on NSAIDs, such as sulindac, aspirin, and celecoxib.

#### 4.2.1. Sulindac

Sulindac, a less selective cyclooxygenase inhibitor, has long been prescribed to FAP patients with colorectal polyps. In the first randomized controlled trial, which was carried out in 1993 by Giardiello et al., 22 patients with FAP were randomly assigned to receive sulindac 150 mg or placebo twice daily for 9 months [63]. Data revealed a 56% decrease in polyp count and a 65% reduction in polyp size in patients using sulindac. However, no patient achieved complete polyp clearance, and the number and size of polyps increased after 3 months of discontinuing sulindac, suggesting the necessity for ongoing treatment.

Subsequently, Giardiello et al. carried out another randomized study with 41 patients who had the APC mutation, but the findings revealed that sulindac at regular doses did not stop the progression of adenomas [64]. The focus of subsequent research moved to COX-2 inhibitor (celecoxib) and its critical contribution to the development of gastrointestinal polyps.

#### 4.2.2. Celecoxib

Colonic adenomas have elevated COX-2 expression, which indicates a higher likelihood of malignant transformation [65]. In the beginning, Steinbach et al. discovered that the polyp burden in patients with FPA was lower in the celecoxib group (400 mg daily) compared to the placebo group (*p* = 0.001) and that there was no difference in adverse events between the groups [66]. Similarly, Phillips et al. discovered further qualitative improvement and quantitative reduction in duodenal polyposis when patients received celecoxib 400 mg twice daily [67]. However, the duration of these studies was short. To solve this problem, Burke et al. performed a study to assess the efficacy of celecoxib in kids with FAP over a 5-year period. The findings demonstrated that fewer patients in the celecoxib group (12.7% vs. 25.5%) reached the primary endpoint (≥20 polyps or >2 mm in size or a diagnosis of CRC) [68]. Celecoxib, the first chemopreventive medicine approved by the Food and Drug Administration (FDA) for the treatment of colonic polyps in patients with FAP, seems to be safe and effective based on the studies mentioned above. However, drug development was eventually halted due to unacceptable cardiovascular toxicity in the general population [69,70].

#### 4.2.3. Aspirin

Since aspirin can simultaneously inhibit the activity of COX-1 and COX-2, it has been a research hotspot. Evidence suggests that aspirin lowers the risk of CRC in both FAP patients and the general population. To determine the efficacy of aspirin (600 mg/d) and/or resistant starch (30 g/d) in treating young persons with FAP, the Concerted Action Polyp Prevention (CAPP) group undertook a multicenter, randomized, controlled trial (CAPP1). The results showed that patients taking 600 mg of aspirin daily tend to have lower polyp burden (both in terms of size and number), but resistant starch does not seem to have any clinical effect on adenomas [71]. In the subsequent CAPP2 study, researchers discovered that giving patients with LS 600 mg of aspirin daily for 25 months significantly decreased the risk of CRC [72]. Ait Ouakrim et al. observed similar results, indicating that LS patients who used aspirin had a lower risk for both 1 month–4.9 years (HR, 0.49; 95% CI, 0.27–0.90; *p* = 0.02) and 5 years (HR, 0.25; 95% CI, 0.10–0.62; *p*= 0.003), compared with less than 1 month of use [73]. The American College of Gastroenterology advises aspirin use for patients with LS to prevent CRC based on the aforementioned research; however, the ideal dosage is yet unknown. Because of this, the CAAPP3 study is being carried out to examine various trial dosages of aspirin (100, 300, or 600 mg daily) in patients with LS, which may eventually provide more conclusive evidence.

#### 4.2.4. Combination Therapy

Combination therapy might be a way to deal with the issue of dose-related side effects. Meyskens et al. demonstrated that the combination of sulindac 150 mg and difluoromethylornithine (DMFO) 500 mg can lower the risk of recurrent adenomatous polyps with few side effects [74]. Similar to this, it has been demonstrated that in adult FAP patients, the combination of sulindac 150 mg/DMFO 750 mg daily is more beneficial than placebo/DMFO 750 mg daily or placebo/sulindac 150 mg daily [75]. Lynch et al. showed that FAP patients with celecoxib/DMFO experienced more significant declines in adenoma burden (32% vs. 22%, *p* = 0.17) and polyp count (11% vs. 1%, *p* = 0.76) than celecoxib monotherapy [76]. In addition, a double-blind, randomized, placebo-controlled research found that 75 mg of erlotinib once a day and 150 mg of sulindac twice a day dramatically reduced the number of duodenal polyps in FAP patients [77].

#### 4.2.5. Other Agents

Certain free fatty acids(FFA) were also linked to a decrease in arachidonic acid levels and COX-2 expression. West et al. discovered a 22.4% reduction in the number of polyps (*p* = 0.012) and a 29.8% reduction in polyp size (*p* = 0.027) in FAP patients treated with EPA-FFA compared to the placebo group, with results similar to those in the COX-2 inhibitor study [78]. The efficacy of Curcumin on the mean number and size of adenomas in FAP patients was also evaluated; however, there was no significant difference compared to the placebo [79]. Furthermore, four patients with FAP received six months of sirolimus treatment, showing promising effects, especially on the number of polyps in the rectal remnant and ileal pouch, although at the cost of numerous adverse events [80]. Ascorbic acid has also been studied because of its antioxidant and antitumor properties [81,82]. Nevertheless, no studies have convincingly demonstrated the clinical benefit of ascorbic acid in reducing the risk of CRC in patients with FAP.

Overall, the optimal chemopreventive agent has not been found in patients with hereditary CRC syndromes, and further exploration of novel chemoprevention strategies is still required.

### 4.3. Immuno-Prevention in Hereditary CRC Syndromes

In recent years, with a better understanding of the mechanisms of tumor immune response, immunotherapy has exploded in the field of oncology treatment, and more precise therapeutic strategies have been developed. CRC with DNA dMMR is characterized by TMB, which can produce abundant neoantigens, thereby activating the antitumor immune response. On the one hand, well-established approaches, namely immune checkpoint blockade, have been developed to generally support the host’s antitumor response capacity. On the other hand, tumor cell-specific or overexpressed neoantigens have opened up prospects for vaccine development.

#### 4.3.1. Immune Checkpoint Inhibitors (ICIs) Therapy

ICIs are a specific type of immunotherapy that can improve antitumor immune responses by inhibiting T-cell negative regulatory receptors, such as cytotoxic T lymphocyte antigen 4 (CTLA4) and programmed cell death 1 (PD-1). ICIs have been demonstrated to be effective among patients with metastatic CRC with MSI-H and/or dMMR. Compared to traditional chemotherapy, ICIs can produce longer progression-free survival with fewer adverse events. An anti-CTLA-4 antibody (ipilimumab) and two anti-PD-1 antibodies (pembrolizumab and nivolumab) were approved by US FDA for the treatment of patients with MSI-H or dMMR metastatic CRC in 2011 and 2014, respectively [83]. Furthermore, clinical trials have repeatedly shown that inhibiting immune checkpoints with pembrolizumab ornivolumab) are effective in treating MSI cancers originating from the colorectum and other organs [83,84,85,86,87]. MSI-H and dMMR are associated with highly immune infiltrative tumor microenvironments, and both of them are the features of LS. Hence, ICIs are being studied as immune-preventive agents in LS [88]. POLE-mutant CRCs show increased CD8^+^ lymphocyte infiltration and high TMB, which are associated with ultramutated phenotype. Thus, CRC patients with POLE alterations may also benefit from immunotherapy [89,90]. As a result of these advancements, hereditary CRC syndromes have emerged as a central focus in the field of precision medicine and immune-oncology in the past years.

#### 4.3.2. Vaccines

The responsiveness to ICIs is determined by several important parameters, such as the TMB (high amounts of tumor neoantigens), tumor-infiltrating lymphocytes, and regulatory checkpoint receptors [91]. Certain tumors have lower T-cell infiltration than others. This could be because of deficiencies in priming or the absence of high-affinity T cells. At this time, vaccinations may prove to be beneficial solutions in combination with ICIs, because they target particular mutant antigens. In contrast to the mostof other tumor types, LS-associated dMMR cancers produce mutational neoantigens and constitute a substantial pool of prospective vaccine targets [92]. These factors will help researchers better understand how vaccines affect patients’ prognoses and survival rates. Dendritic cells (DC) known as the strongest antigen-presenting cells play a key role in initiating the immune system’s response to exogenous antigens. Thus, DC vaccine adjuvant can stimulate DC antigen submission, which can induce therapeutic CD4^+^T-cell and CD8^+^T-cell responses and facilitate the conversion of naive T cells to cytotoxic T cells [93].

In addition, several clinical and pre-clinical trials for LS and dMMR cancer are underway to test the safety and immunogenicity of frameshift peptide (FSP) neoantigen-based vaccine [94,95,96]. In a phase I/Iia clinical trial, 22 patients with advanced dMMR CRC received FSP neoantigen-based vaccine, and the results showed that vaccine-induced humoral and cellular immune responses were observed in all patients and no vaccination-induced severe adverse events occurred [95]. Thus, vaccines may be a promising novel approach for the treatment and even prevention of dMMR cancer, even though it is necessary to evaluate vaccines using more appropriate molecular markers, larger randomized trials, and more patients with early-stage CRC, which will be the goal researchers are working towards.

## 5. Conclusions

Hereditary CRC syndromes are a prominent example of how our knowledge of the human disease has advanced over the past 30 years. Effective targeted therapies are now available for some of the affected signaling pathways, such as the NSAIDs sulindac and celecoxib that significantly reduce adenoma load in patients with *APC* germline mutations, probably because of the typical inhibition of the WNT/β-catenin signaling pathway. In addition, breakthroughs in LS vaccination have been achieved using FSP neoantigens induced by dMMR mechanisms. Nevertheless, additional research is required to improve our understanding of the genotype–phenotype variety of hereditary CRC syndromes as well as the most effective management strategies. The cost of conducting genetic research techniques is continuing to decline, which is pushing the concept of precision medicine closer and closer to being a reality.

## Figures and Tables

**Table 1:** Risk of hereditary colorectal cancer syndromes.

Cancer Syndromes	CRC Lifetime Risk	Average Age of CRC Diagnosis	Age of Screening Initiation	Intervals of Screening	Incidence in CRC Population
Sporadic	4.3%	67	50	10	65–85%
FAP (AFAP)	100% (70%)	40 (55)	10–15	1	~1%
MAP	43–100%	48	20–25	1–3	<1%
JPS	39%	44	15	1–3	<1%
PJS	39%	42–46	15	2–3	<1%
PHTS	9–18%	35	35	5	<1%
LS	50–80%	45	20–25	1–2	2–3%

Note: FAP, familial adenomatous polyposis; AFAP, attenuated familial adenomatous polyposis; MAP, MUTYH-associated polyposis; JPS, juvenile polyposis syndrome; PJS, Peutz–Jeghers syndrome; PHTS, PTEN-hamartoma tumor syndrome; LS, Lynch syndrome.
